# Competing risks data in clinical oncology

**DOI:** 10.3389/fonc.2024.1360266

**Published:** 2024-04-04

**Authors:** Haesook Teresa Kim

**Affiliations:** Department of Data Science, Dana-Farber Cancer Institute, Boston, MA, United States

**Keywords:** competing risks, data analysis, clinical utility, cancer treatment, efficacy

## Abstract

Competing risks data analysis plays a critical role in the evaluation of clinical utility of specific cancer treatments and can inform the development of future treatment approaches. Although competing risks data are ubiquitous in cancer studies, competing risks data are infrequently recognized and competing risks data analysis is not commonly performed. Consequently, efficacy of specific treatments is often incompletely and inaccurately presented and thus study results may be interpreted improperly. In the present article, we aim to enhance awareness of competing risks data and provide a general overview and guidance on competing risks data and its analysis using cancer clinical studies.

## Introduction

Advances in early detection and development of innovative cancer treatments have substantially reduced cancer death rates and improved overall survival (OS) (1, 2). Although OS is the most unambiguous metric for assessing efficacy of cancer therapy, determination of OS requires much longer follow-up in many cancers and may be confounded by the effect of salvage therapies used following disease recurrence (3). For these reasons, composite endpoints that combine death and non-fatal events such as disease recurrence or institution of new therapy have been widely adopted to assess the cancer treatment effect. Commonly used composite endpoints include disease-free survival (DFS), progression-free survival (PFS), or event-free survival (EFS). By combining several clinically relevant endpoints into an aggregated single endpoint, a composite endpoint is simple and can substantially reduce sample sizes needed to compare treatment strategies. Although composite endpoints are excellent measures of overall effectiveness, it assumes that an intervention benefits all of the component outcomes in the same direction (4), and this assumption is often not met in cancer clinical trials. For example, more intense treatment is often associated with a decreased risk of disease recurrence but also with an increased risk of treatment-related morbidity and mortality.

Competing risks data are ubiquitous in cancer studies and occur across all types of cancer (5). Although competing risks data analysis plays a critical role for determining clinical utility of cancer treatment, competing risks are often not acknowledged and competing risks data analysis is not commonly performed (6). Consequently, the effectiveness of a treatment is often incompletely presented and thus study results may be interpreted improperly. In the present article, we aim to enhance awareness of competing risks data and provide a general overview and guidance on competing risks data and its analysis. We do not describe development of different statistical methods and issues (7–11), competing risks data analysis (12–14) and overview of competing risks data (15, 16) as information on these topics has been well described elsewhere. Rather, we focus on two parts of analysis using commonly used statistical methods. One is estimation of cumulative incidence (CumInc) function of each event, and the other is competing risks regression analysis. In the sections to follow, we present the definition of competing risks, types of competing risks data, recognition of competing risks data, and competing risks data analysis using examples of clinical cancer studies.

## Types of competing risks

Competing risks occur when failure (or event) can be classified by its types (5). Competing risks can be categorized as *classic* or *semi-competing risks* (17–19). The *classic* competing risks problem arises when there are mutually exclusive events and the occurrence of one type of event prevents the occurrence of other types of events. The *semi-competing* situation arises when the occurrence of an event of interest may be prevented by the occurrence of competing events but not vice versa. Typical classic competing risks in cancer studies are disease recurrence or death due to disease (termed as *disease recurrence or relapse*) and death due to treatment-related toxicity (termed as *treatment-related mortality, TRM*). Of note, the term *non-recurrence mortality* or *non-relapse mortality* (NRM) is also used in place of TRM to be more inclusive of any non-recurrence deaths whether these deaths are directly related to treatment or not. The two terms TRM and NRM are often used interchangeably. Here, both disease recurrence and TRM are considered to be terminal events. In this context, disease recurrence is an indication of treatment failure and thus regarded as a terminal event even if a patient responds to a new intervention following disease recurrence. Examples of semi-competing risks are death and serious infection after cancer treatment or graft-versus-host-disease (GVHD) after allogeneic hematopoietic cell transplantation (alloHCT). In this context, infection or GVHD are not considered to be terminal events because while death prevents the occurrence of infection or GVHD, the occurrence of these events does not prevent occurrence of death. This is distinct from censoring because regardless of the duration of follow-up, the outcome of interest, infection or GVHD, will never occur for those subjects who die without infection or GVHD. In classic competing risks, both events are typically events of interest whereas in the semi-competing risks setting, one event (non-terminal) is an event of interest and the other one (i.e., death) is a competing event.

### Recognition and presentation

**Figure 1:**
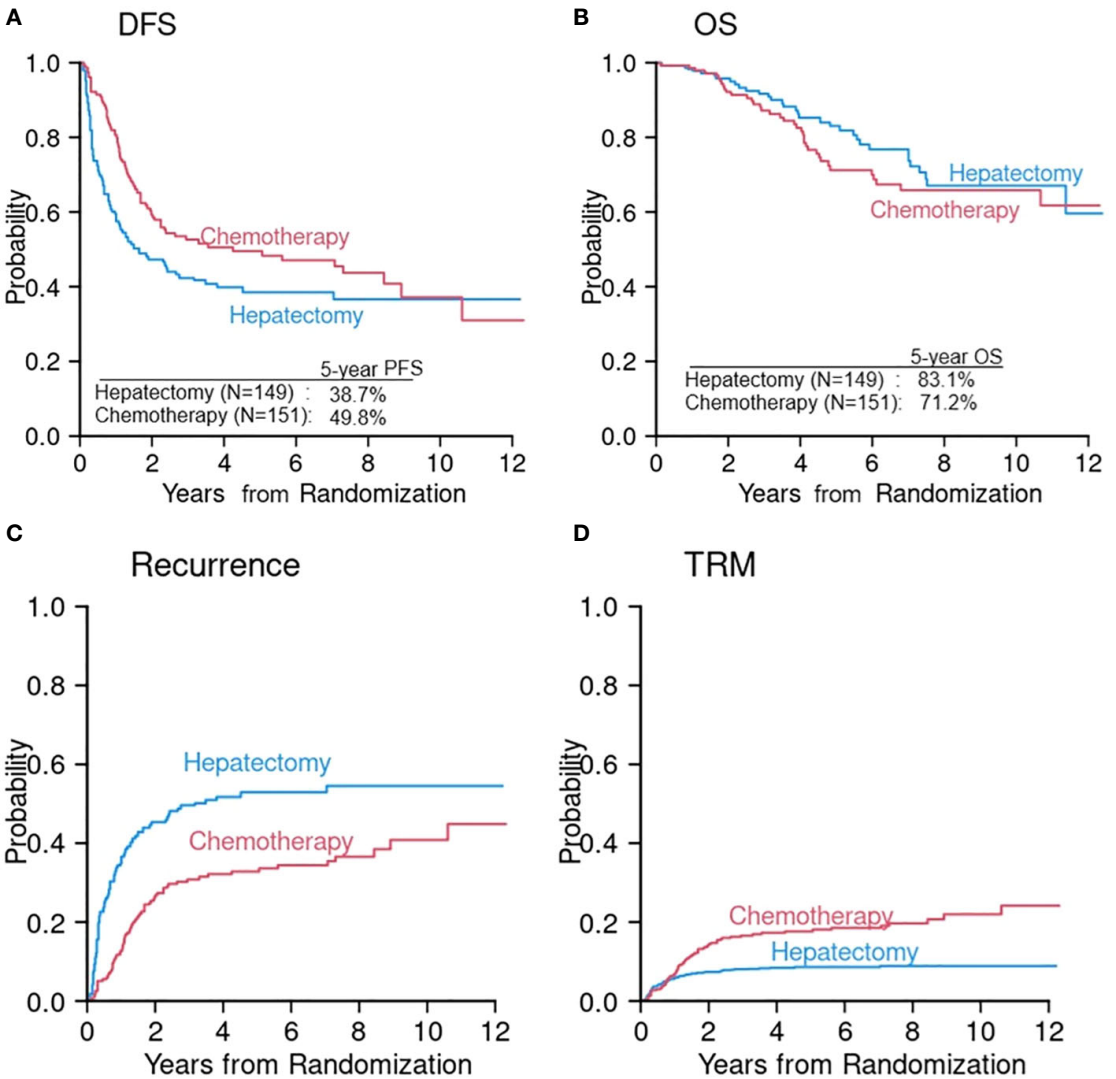
Reconstruction of DFS **(A)** and OS **(B)** from a randomized phase III study of hepatectomy alone (hepatectomy arm) versus hepatectomy followed by chemotherapy (chemotherapy arm) (20). Cumulative incidences of disease recurrence **(C)** and TRM **(D)** were constructed from the information extracted in the original article (20).

The primary reason for underusage of competing risks data analysis is lack of recognition and lack of understanding its value. We illustrate this point using recently published data (20). In a randomized controlled trial of adjuvant modified fluorouracil, leucovorin, and oxaliplatin (mFOLFOX6) after hepatectomy (chemotherapy arm) compared with hepatectomy alone (hepatectomy arm) in liver-only metastatic colorectal cancer, 5-year DFS was 49.8% in the chemotherapy arm and 38.7% in the hepatectomy arm (p = 0.006) (**Figure 1A**) (20). The trial was terminated early according to the protocol because DFS was significantly longer in the chemotherapy arm. However, 5-year OS was lower in the chemotherapy arm (71.2%) compared with the hepatectomy arm (83.1%) (**Figure 1B**). In the chemotherapy arm, more than 50% of patients experienced chemotherapy related grade 3 or higher toxicity suggesting that although the adjuvant chemotherapy may prevent disease recurrence, it also comes with an increased risk of toxicity, which may be attributable to the additional chemotherapy. Despite conflicting results between DFS and OS, the paper did not further investigate the component events of DFS—namely, disease recurrence and TRM. It did not compare CumInc of these two critical events between two arms in a *post-hoc* analysis, suggesting that competing risks were not recognized. It reported, however, that during follow-up, the crude recurrence rate was 56% in the hepatectomy arm and 45% in the chemotherapy arm. Since the recurrence rate is lower but the OS is also lower in the chemotherapy arm, one may speculate that the NRM rate might be higher in the chemotherapy arm compared with the hepatectomy arm. To investigate competing risks further, we first reproduced DFS and OS using digital software to read in the coordinates of the Kaplan–Meier (KM) curves and reconstructed the KM curves according to the method proposed by Guyot at el (21)(**Figures 1A, B**). We then projected CumInc of recurrence and TRM (**Figures 1C, D**). In this projection, we assumed that the recurrence rate is a proportion of one minus DFS since one minus DFS is the sum of disease recurrence and TRM at each time point. The proportion in each arm was calculated as the number of recurrence divided by the total number of events. This information was provided in the original article (20). TRM is then one minus DFS minus recurrence rate. Of note, these CumInc curves are hypothetical and may be different from the real data. The purpose of this graphical display is to show that additional analysis of competing risks data can reveal more detailed information about the treatment efficacy and guide the direction of future treatment.

In another example, a randomized, double-blind, placebo-controlled phase 3 trial in early triple-negative breast cancer (22) reported that the primary endpoint, EFS, was significantly higher in the pembrolizumab-chemotherapy arm compared with the placebo-chemotherapy arm (3-year EFS 84.5% vs. 76.8%, respectively, p < 0.001). The paper tabulated the frequencies of first events which we converted into a bar chart for visual comparison of all component events in EFS (**Supplementary Figure 1A**). As shown in **Supplementary Figure 1A**, frequencies of distant recurrence and disease progression appear to be lower in the pembrolizumab-chemotherapy arm compared with the placebo-chemotherapy arm, indicating that the additional pembrolizumab treatment affects distant recurrence the most. It would be informative if competing risks data analysis were performed to examine whether pembrolizumab is beneficial to certain events and not all events. **Supplementary Figures 1B, C** are hypothetical curves derived from the frequencies of events.

### Competing risks data analysis

Briefly, much of early theoretical development in competing risks data focused on a set of latent failure times and cause-specific hazard that can be formulated as the marginal distributions of latent failure times (7–9). However, because these latent failure times are hypothetical and unobservable, the marginal distribution is unidentifiable unless independence is assumed between competing risks (10). For example, if a subject dies of disease recurrence, time to TRM (competing event) for this patient is unobservable (i.e., latent) because the event of TRM can no longer occur after the subject dies. If recurrence and TRM are independent, conventional survival analysis can be performed for each event. However, the independence assumption is untestable and unjustifiable. A CumInc function, on the other hand, is often more appealing and circumvents the unidentifiability issue.

**Figure 2:**
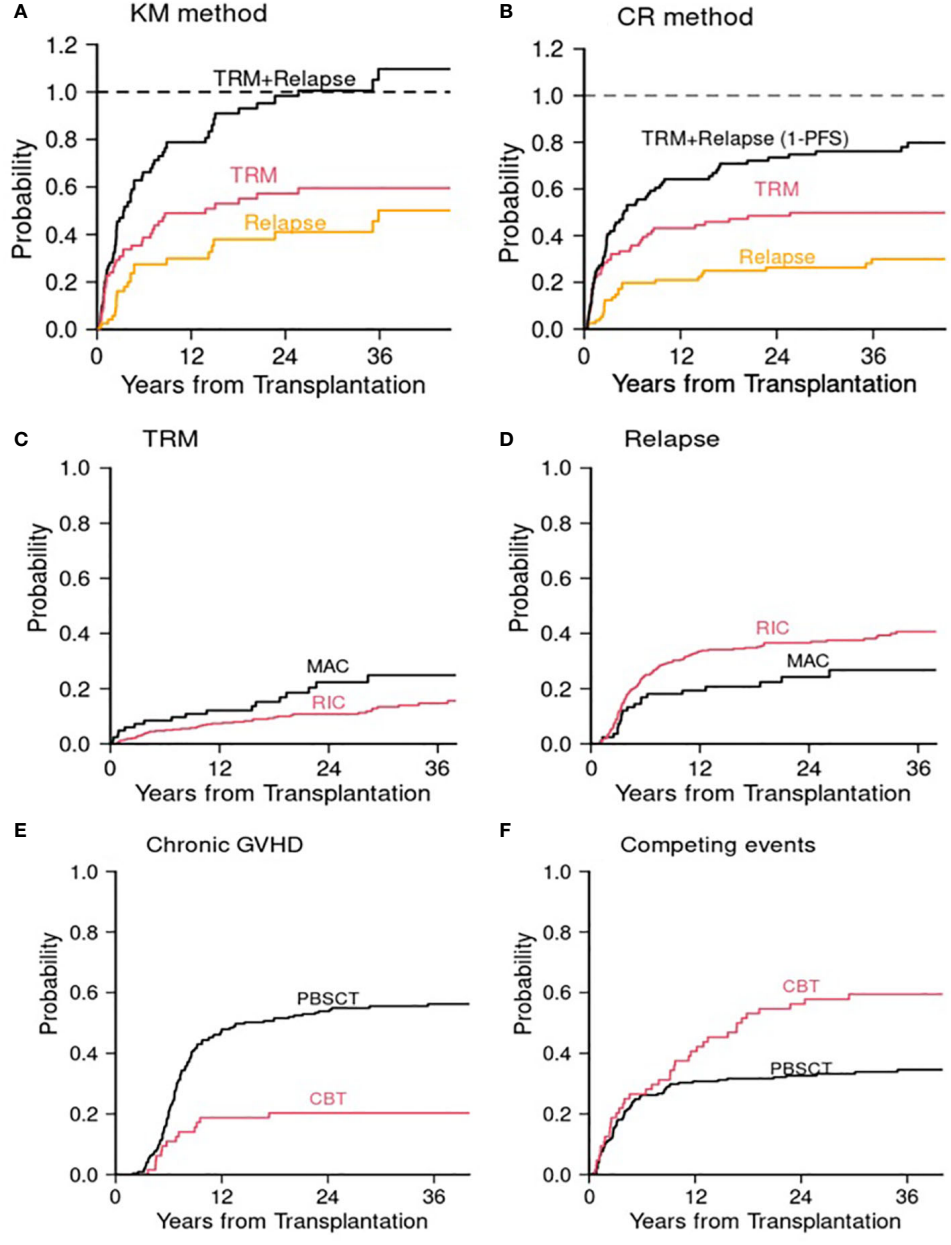
Allogeneic transplant studies. Cumulative incidences of TRM and relapse in the MAC cohort (23) using **(A)** the Kaplan–Meier (KM) method and **(B)** competing risks (CR) method. **(C)** and **(D)** Post transplant cyclophosphamide study (24). **(C)** Cumulative incidence of TRM for MAC and RIC. **(D)** Cumulative incidence of relapse for MAC and RIC. **(E)** and **(F)** Cord blood transplant study (25). **(E)** Cumulative incidence of chronic GVHD. **(F)** Cumulative incidence of competing events (death or relapse without developing chronic GVHD). MAC, myeloablative conditioning; RIC, reduced intensity conditioning; CBT, cord blood transplantation; PBSCT, peripheral blood stem cell transplantation. The Gray test was used for the comparison of cumulative incidence.

In parallel with standard survival analysis, competing risks data analysis is composed of two parts: one is estimation of CumInc of competing events, and the other is competing risks regression analysis (5). Just as estimation of single endpoints (e.g., OS, PFS) using the KM method in standard survival analysis, competing risks events are typically presented with estimation of CumInc function of an event of interest in the presence of competing risks. Detailed description of estimation was previously reported (5). Essentially, there are two ways to estimate CumInc function of an event. One is using the KM method that ignores competing events, and the other is to estimate the CumInc function in the competing risks framework (5). Because the KM method ignores competing events by using the cause specific survival estimate, which censors competing events, at each time point, this method overestimates the CumInc. To illustrate the difference of these two methods, we used the previously published data of comparing outcome between reduced intensity conditioning (RIC, N = 71) and myeloablative conditioning (MAC, N = 81) for patients older than 50 years undergoing alloHCT (23). If the KM method is used for 81 patients who received MAC alloHCT, the 3-year CumIncs of TRM and relapse were 58% and 50%, respectively. Even though some patients were still alive and disease-free at 3 years and these two events are mutually exclusive, the sum of these two events is 108% at 3 years (**Figure 2A**). If, however, it was estimated in the competing risks framework, the 3-year CumIncs of TRM and relapse were 50% and 30%, respectively (**Figure 2B**). Thus, the combined rates at 3 years is 80% which is one minus 3-year PFS. The KM method overestimated TRM by 8% and relapse by 20%. The magnitude of overestimation depends on the incidence rate, onset timing of competing events, and interdependency of two events. In this example, because many TRM events occurred early in MAC, the CumInc rate of relapse was affected more significantly because cause-specific relapse-free survival estimate was high at later time points in the KM method as early TRM cases were treated as censored observations. This example shows that ignoring competing risks may cause a significant consequence of overestimation of the CumInc rate of the event of interest and the interpretation of results.

### Competing risks regression analysis

Because composite endpoints combine all failure events together, it is not possible to identify risk factors for each individual component event. Particularly, risk factors for directionally opposing component events may not be identified. For example, if a treatment reduces disease recurrence but increases TRM, the influence of this treatment on TRM or recurrence may not be identified in the assessment of risk factors for PFS due to the opposing effects of two events. Therefore, it is difficult to make any meaningful interpretation of the result from multivariable regression analysis for PFS. To illustrate competing risks regression analysis and the effect of risk factors on outcomes, we used the recently published alloHCT data (24). In this analysis, we employed two most commonly used regression methods for competing risks data. These are the cause-specific hazard regression using Cox model (7) and the subdistribution hazard regression proposed by Fine and Gray (26). For detailed information of these methods, we refer to previous papers (7, 26). Briefly, in the cause-specific hazard regression using the Cox model, failures from other causes are treated as censored observations and the effect of covariates is on the instantaneous probability of failing from a cause of interest given a subject experienced no event until time t (cause-specific hazard). This analysis is identical to performing a standard Cox regression analysis for a single type of failure. Since the simple relationship between a single endpoint and a single cause-specific hazard does not hold in the presence of competing risks, Fine and Gray (26) proposed a direct regression modeling of the effect of covariates on the CumInc function for competing risks data. Because these two approaches were constructed from different stochastic quantities, the results from these approaches should be interpreted differently and appropriately.

In a retrospective analysis examining the effect of posttransplant cyclophosphamide as a GVHD prophylactic regimen for patients age 50 or older, less intense RIC was significantly associated with a lower TRM risk (subdistribution hazard ratio (sHR) 0.57, p = 0.04) compared with intense MAC but with a higher disease recurrence risk (sHR 1.71, p = 0.026) (**Supplementary Table 1A**; **Figures 2C, D**). Since the effect of conditioning intensity (RIC vs. MAC) is directionally opposing, when these two events were combined in PFS, the conditioning intensity was not significant (HR 1.16, p = 0.4). (**Supplementary Table 1A**) The cause-specific hazard ratios using Cox model were similar to those of sHRs, but attenuated for TRM (cause-specific hazard ratio (cHR) 0.63, p = 0.1 for TRM, cHR 1.62, p = 0.044 for disease recurrence). Age was significant for PFS (HR 1.5, p = 0.028). More granular competing risks analysis revealed that age ≥60 was associated with increased risk of disease recurrence (sHR 1.68, p = 0.019; cHR 1.65, p = 0.025) but not with TRM (**Supplementary Table 1A**). Male sex was also significantly associated with PFS (HR 1.44, p = 0.009). More detailed competing risks analysis revealed that it was associated with both TRM and disease recurrence in the same direction. However, the association was significant with TRM but not with disease recurrence. When the effects of component events point in the same direction, the total event size is split between two events and thus each effect may get attenuated. This example demonstrates effects of covariates under different scenarios using two regression methods: when a covariate affects one event only and when a covariate affects both events either in the same direction or in the opposite direction. In this example, results from two regression methods are largely concordant except when the effect of a covariate opposes.

Another example is a comparison between cord blood transplantation (CBT) and unrelated donor peripheral blood stem cell transplantation (PBSCT) (25). In this retrospective data analysis, we observed that CBT was associated with a decreased risk of chronic GVHD (non-terminal event) but with an increased risk of the competing risks, that is, disease recurrence or death without developing chronic GVHD. Of note, the competing risk of a non-terminal event is usually death without the event of interest. However, since patients who develop chronic GVHD typically do not experience disease recurrence (or vice versa), disease recurrence and death without developing chronic GVHD were regarded as competing events in this analysis. This phenomenon is known as a graft-versus-leukemia and GVHD effect, a see-saw effect. In multivariable regression analysis, both sHR and cHR were 0.3 for chronic GVHD for CBT relative to PBSCT. However, the sHR for the competing events was 1.79 (p = 0.006) and cHR was 1.41 (p = 0.10). To shed some light on, CumIncs of chronic GVHD and its competing events are presented in **Supplementary Table 1B** and **Figures 2E, F**. Undoubtedly the CumInc of chronic GVHD was lower in the CBT cohort, but the CumInc of competing events was higher compared with the PBSCT, which is aligned with the result from the Fine and Gray model (**Supplementary Table 1B**). In this example, if one presents the event of interest only (chronic GVHD), one could falsely claim that CBT is preventive of chronic GVHD. However, the low CumInc of chronic GVHD was in part affected by the high incidence of competing events. This is largely due to the fact that many patients died of transplant-related complications such as infection after CBT. This phenomenon was previously observed (27). This example highlights the proper interpretation of results, which requires data analyst to have in-depth knowledge and insight in both disease and competing risks data analysis.

## Discussion

As most cancer treatments come with substantial risks of treatment-related morbidity and mortality, if a composite endpoint such as PFS or EFS, is the primary endpoint, data analysis of the composite endpoint must be accompanied by the analyses of individual component events. Presenting the analysis of a composite endpoint alone may mislead the true efficacy and confound interpretation of cancer treatment strategies. Even when efficacy is demonstrated for the composite endpoint, it is critical to examine the effect of treatment intervention on individual events to obtain more complete interpretation of the results, which will eventually affect patient care decisions. This is particularly important if the effect of a treatment is directionally opposing in component events. If the clinical importance of different component events is substantially different and a component with greater clinical importance (e.g., death or relapse) appears to be adversely affected by the treatment, even if the event of interest with lesser clinical importance (e.g., infection, GVHD) is favorably affected, this is not an indicator of treatment efficacy.

As to the data analysis, since the interdependency, the timing and the magnitude of competing events are unknown *a priori*, the general rule of thumb is to present CumInc curves to gain insight of competing events. Depending on this result, the interpretation should be made in the disease-specific context. The choice of competing event should be disease specific. In general, for the terminal event of interest, all events other than the event of interest can be competing events. For non-terminal events of interest such as infection, death without the event of interest is typically the competing event.

Competing risks regression analysis has been very useful for determining clinical utility of treatment and identifying risk factors for each event. For competing risks regression analysis, cause-specific hazard regression and subdistribution hazard regression have been widely and commonly used due largely to the readily available statistical software packages, such as R (R packages ‘*cmprsk*’, ‘*EzR*’), Stata, and SAS that offer a variety of implemented functions. To examine the difference between these two approaches, Dignam et al. conducted a simulation study under two scenarios: when events are independent and when events are positively correlated (13). Because the direction and the level of dependency, the onset timing, and the frequency of competing events are unknown *a priori*, it may not be fully feasible to simulate the real-world situation accurately. In general, when a covariate influences on one event only, the analysis results from two models are largely concordant. When a covariate influences on both the event of interest and competing events in the opposite direction, detailed investigation including graphical display of CumInc is needed to interpret the analysis result properly. Since the Fine and Gray model was built directly on CumInc function, the result from this model is consistent with the difference of CumInc curves even when the proportionality assumption is not necessarily met. For other regression models, we refer to Haller et al. (28) for an overview over various methods.

## Author contributions

HK: Conceptualization, Data curation, Formal analysis, Investigation, Methodology, Project administration, Supervision, Visualization, Writing – original draft, Writing – review & editing.
