# Supplementary material for: Competing risks data in clinical oncology
Source: Front Oncol. 2024 Apr 4;14:1360266. doi: 10.3389/fonc.2024.1360266 (PMC11024219; doi:10.3389/fonc.2024.1360266)
Supplement: Supplementary file 1 [file DataSheet_1.docx]

**Table S1.** Comparison of Cox regression model and Fine and Gray model for competing risks of TRM and relapse using the post transplant cyclophosphamide study ^23^ (A) and competing risks of chronic graft-versus-host disease (GVHD) and disease recurrence or death without developing chronic GVHD using the cord blood transplantation study.^24^ (B) RIC: reduced intensity conditioning regimen. MAC: myeloablative conditioning regimen. PTCY: post transplant cyclophosphamide graft-versus-host disease prophylactic regimen. CBT: cord blood transplantation. PBSCT: peripheral blood stem cell transplantation. Myeloid disease includes acute myeloid leukemia, myelodysplastic syndrome, chronic myeloid leukemia, and myeloproliferative neoplasms. Lymphoid disease includes acute lymphocytic leukemia, chronic lymphocytic leukemia, lymphoma. HR: hazard ratio. cHR: cause-specific hazard ratio. sHR: subdistribution hazard ratio

1. **Example of post transplant cyclophosphamide study**

|  | PFS | | TRM | | | | Relapse | | | |
| --- | --- | --- | --- | --- | --- | --- | --- | --- | --- | --- |
|  | Cox Model | | Cox Model | | Fine and Gray model | | Cox Model | | Fine and Gray model | |
|  | HR (95% CI) | p-val. | cHR (95% CI) | p-val. | sHR (95% CI) | p-val. | cHR (95% CI) | p-val. | sHR (95% CI) | p-val. |
| Age >=60 vs. <60 | 1.5 (1.05-2.15) | 0.028 | 1.2 (0.64-2.25) | 0.57 | 1.04 (0.55-1.96) | 0.9 | 1.65 (1.06-2.57) | 0.025 | 1.68 (1.09-2.57) | 0.019 |
| Male vs. Female | 1.44 (1.10-1.89) | 0.009 | 1.89 (1.11-3.23) | 0.02 | 1.72 (1.01-2.93) | 0.046 | 1.30 (0.95-1.78) | 0.11 | 1.26 (0.92-1.72) | 0.15 |
| RIC vs. MAC | 1.16 (0.82-1.65) | 0.40 | 0.63 (0.36-1.1) | 0.1 | 0.57 (0.33-0.98) | 0.044 | 1.62 (1.01-2.58) | 0.045 | 1.71 (1.07-2.73) | 0.026 |
| PTCY vs. Other | 0.64 (0.43-0.95) | 0.025 | 0.72 (0.34-1.53) | 0.4 | 0.78 (0.37-1.63) | 0.5 | 0.61 (0.38-0.97) | 0.036 | 0.61 (0.39-0.96) | 0.03 |

1. **Example of cord blood transplantation study**

|  | Chronic GVHD | | | | Competing event (Recurrence/Death) | | | |
| --- | --- | --- | --- | --- | --- | --- | --- | --- |
|  | Cox model | | Fine and Gray model | | Cox model | | Fine and Gray model | |
|  | cHR (95% CI) | p-val | sHR (95% CI) | p-val | cHR (95% CI) | p-val | sHR (95% CI) | p-val |
| CBT vs PBSCT | 0.30 (0.16, 0.53) | <.0001 | 0.30 (0.16, 0.55) | 0.0001 | 1.41 (0.93, 2.15) | 0.10 | 1.79 (1.18, 2.73) | 0.006 |
| Age>=50 vs <50 | 1.66 (1.05, 2.64) | 0.032 | 1.73 (1.10, 2.74) | 0.019 | 1.09 (0.69, 1.71) | 0.72 | 0.94 (0.59, 1.49) | 0.79 |
| Myeloid vx. Lymphoid | 0.76 (0.52, 1.11) | 0.1556 | 0.56 (0.38, 0.81) | 0.002 | 1.28 (0.85, 1.93) | 0.23 | 1.45 (0.98, 2.13) | 0.06 |

**Figure S1.** Components of event-free survival from a phase 3 trial with pembrolizumab versus placebo in early triple-negative breast cancer^23^. PD: progression of disease that precluded definitive surgery. Local R: local recurrence. Distant R: distant recurrence. Second M: second primary malignancies.

1. Frequency of events

**
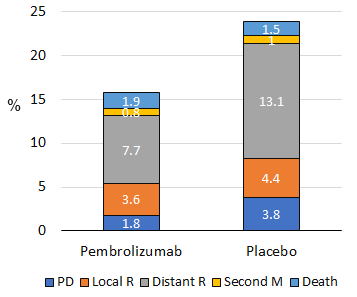
**

**
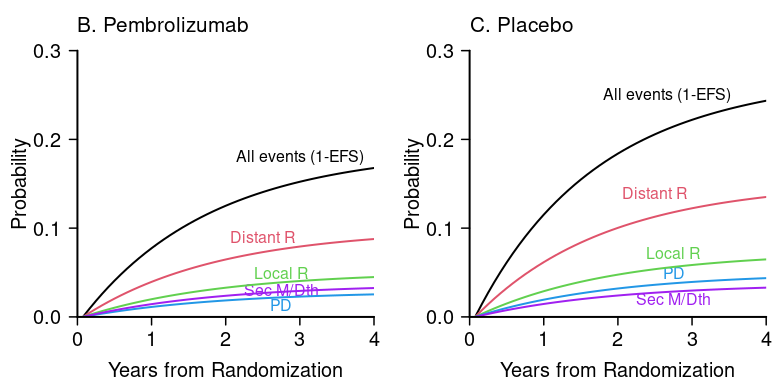
**
